# Characterization of carbapenemase-producing *Enterobacterales* from rectal swabs of patients in the intensive care units of a tertiary hospital in Cali-Colombia

**DOI:** 10.1016/j.heliyon.2024.e33368

**Published:** 2024-06-21

**Authors:** Mónica Fernandes-Pineda, Ernesto Martínez-Buitrago, José H. Bravo, Lorena Matta-Cortés, Johann A. Ospina-Galindez, Claudia C. Paredes-Amaya

**Affiliations:** a Department of Internal Medicine, Faculty of Health, Universidad del Valle, Cali, Colombia; b Department of Medical Sciences, Faculty of Health Sciences, Pontificia Universidad Javeriana Cali, Cali, Colombia; c Faculty of Engineering, Universidad Autónoma de Occidente, Cali, Colombia; d Department of Microbiology, Faculty of Health, Universidad del Valle, Cali, Colombia

**Keywords:** Carbapenemase-producing *Enterobacterales*, Antibiotic resistance, Carbapenem-resistant, *Klebsiella pneumoniae*

## Abstract

**Background:**

Carbapenemase-producing *Enterobacterales* (CPE) represents a significant threat to global health. This study aimed to characterize clinically and molecularly the CPE isolated from rectal swabs of patients in the intensive care units (ICUs) of a tertiary hospital in Cali, Colombia.

**Methods:**

This was a cross-sectional observational study. Rectal swabs from patients admitted to the ICUs were collected. Bacterial identification and carbapenemase production were determined using phenotypic and molecular methods. Demographic and clinical data were extracted from electronic medical records.

**Results:**

The study included 223 patients. Thirty-six patients (36/223, 16.14 %) were found to be colonized or infected by CPE. Factors such as prolonged stay in the ICU, previous exposure to carbapenem antibiotics, use of invasive procedures, and admission due to trauma were associated with CPE. *Klebsiella pneumoniae* (52.5 %) was the most prevalent microorganism, and the dominant carbapenemases identified were KPC (57.8 %) and NDM (37.8 %).

**Conclusion:**

Distinguishing carbapenemase subtypes can provide crucial insights for controlling dissemination in ICUs in Cali, Colombia.

## 1 Introduction

Carbapenemase-producing *Enterobacterales* (CPE) are among the most significant public health challenges worldwide. Infections caused by CPE lead to more extended hospital stays, increased healthcare costs, and higher mortality rates than infections caused by carbapenem-susceptible isolates [1,2]. Since the first isolation of carbapenem-resistant *Klebsiella pneumoniae* in 1996, carbapenemases have become a major public health concern [3]. Carbapenemases are enzymes that hydrolyze beta-lactam antibiotics, including carbapenems. Several types of enzymes have been described over the years like *Klebsiella pneumoniae carbapenemase* (KPC), New Delhi metallo-betalactamase (NDM), Verona Integron-encoded Metallo-betalactamase (VIM), Imipenemase metallo-betalactamase (IMP), and Oxacillinase (OXA)-48, which are the most relevant types in clinical settings [4,5]. In Colombia, KPC-producing bacteria began to spread in the late 2000s [6].

The prevalence of CPE in Colombia varies widely, with reported rates of 1 % for *Escherichia coli*, 23 % for *K. pneumoniae*, and 60 % for *Providencia rettgeri* [7]. The clinical relevance of these bacteria mainly lies in the severe infections they can cause [8]. According to data published by the National Institute of Health in Colombia, around 10 % of CPE and carbapenemase-producing *Pseudomonas aeruginosa* express more than one distinct class of these enzymes simultaneously [9]. This represents a significant diagnostic challenge since the phenotypic tests used in most Colombian laboratories are unable to detect these co-productions [10].

Expanding globally, KPC has become endemic in South America, with Colombia emerging as a notable hotspot [11]. This widespread dissemination of multidrug-resistant (MDR) Gram-negative rods (GNR), especially *Enterobacterales* and non-fermentative rods like *Pseudomonas* spp. and *Acinetobacter* spp.*,* when associated with any infection, serves as a significant contributing factor to increased healthcare costs, prolonged hospital stays, and elevated mortality rates [10,12]. It remains an enduring challenge for the healthcare system.

In Colombia, there are few reports on the genetic characterization of CPE, especially in intensive-care adult units (ICUs), and it is unclear whether they are clonally related by descent [9]. The aim of this study was to characterize clinically and molecularly the CPE isolated from rectal swabs of patients in the ICUs of a tertiary hospital in Cali, Colombia.

## 2 Materials and methods

### 2.1 Study population and data collection

This cross-sectional observational study was conducted in ICUs of a tertiary public hospital in Cali, Colombia, between July 2022 and April 2023. Patients aged 18 years or older who stayed at least 48 h in any of the six ICUs were enrolled after providing consent. Those who did not meet these criteria or had incomplete medical history data were excluded. Patients with mental disabilities and who could not provide consent were also excluded.

Demographic and clinical data, including age, sex, date of ICU admission, comorbidities, admission diagnosis, use of invasive medical devices, prior exposure to antibiotics (with a focus on carbapenems), and the primary focus on infection were collected from electronic records.

### 2.2 Sampling

Rectal swabs were obtained in duplicate from all the participants. One swab was used to isolate *Enterobacterales* bacteria on solid media culture. The other swab was stored at 4 °C for a maximum of 72 h until culture results and identification were obtained. If the culture result was positive for CPE, the second swab was used for molecular analysis.

### 2.3 Bacterial and carbapenemase identification

Rectal swabs were cultured on CHROMID CARBA agar for presumptive identification of carbapenemase-producing *Enterobacterales*. Then, bacterial identification and antimicrobial susceptibility tests were performed using the BD Phoenix™ M50 automated system. The presence of carbapenemases in the bacteria was confirmed using the phenotypic Carba NP test according to the manufacturer's instructions [13]. Carbapenemase-positive was defined as all *Enterobacterales*, in which the phenotypic Carba NP confirmed the presence of carbapenemases. After identification, the bacteria were preserved in lysogeny broth with 30 % glycerol at −80 °C. Further, lateral flow and molecular tests were employed to identify the specific types of carbapenemases (KPC, VIM, IMP, NDM, and OXA-48) [14]. The lateral flow test was performed on CPE bacterial colonies, and for molecular identification, the Xpert-Carba R assay was performed on rectal swabs. Only rectal swab samples that tested positive for CPE by phenotypic methods were utilized for molecular analysis.

### 2.4 Data analysis

A descriptive analysis was conducted on demographic and clinical variables, yielding central tendency and dispersion measures for continuous data and frequencies for categorical ones. When comparing the CPE positive and negative groups, suitable statistical tests were applied based on the nature of the data, using the Chi-squared test or Fisher's exact test as appropriate. Odds ratios (OR) with 95 % confidence intervals were calculated to ascertain the strength and direction of associations between categorical variables, setting a significance threshold of 5 %. The findings informed the creation of descriptive and comparative graphics using the ggplot2 library in R, offering a visually intuitive data interpretation. The entire statistical analysis was performed in R, upholding a significance level of 5 % for statistical evaluations.

## 3 Results

### 3.1 Clinical and demographic characteristics

The study included 223 patients admitted to the adult intensive care units of a single tertiary public hospital between July 2022 and April 2023. Thirty-six patients (16.14 %, 36/223) tested positive for carbapenemase-producing *Enterobacterales* (CPE). Among these patients, eight had invasive infections with sepsis (22.22 %), while three had localized infections (8.33 %). The distribution of admission diagnoses was as follows: malignancy (n = 8, 22.22 %), sepsis (n = 8, 22.22 %), gastrointestinal disease (n = 5, 13.89 %), cardiovascular disease (n = 4, 11.11 %), pneumonia/lung disease (n = 4, 11.11 %), trauma (n = 3, 8.33 %), localized infection (n = 3, 8.33 %), and one case of neurological disease (2.78 %) (Table 1).

**Table 1:** Clinical and demographic characteristics of the patients at the time of admission in the ICUs.

Variable	CPE positive (n = 36)	CPE negative (n = 187)	OR (95 % IC)	*p value*
**Sex (M)**	25 (69.44 %)	95 (50.80 %)	2.18 (1.031–4.88)	0.0412
**Mean age (years) (±SD)**	49.6 (±19.0)	54.9 (±20.4)		0.1944
**ICU stay (±SD)**	22.5 (±16.2)	15.6 (±12.9)		0.0002
**Mortality**	6 (16.66 %)	19 (10.16 %)	1.79 (0.600–4.679)	0.2770
**Admission diagnostic**				
Diabetes mellitus	0 (0 %)	3 (1.6 %)	–	
Cardiovascular disease	4 (11.11 %)	25 (13.37 %)		
Gastrointestinal disease	5 (13.89 %)	11 (5.88 %)	0.364 (0.073–1.687)	0.1945
Neurological disease	1 (2.78 %)	19 (10.16 %)	2.728 (0.342–78.914)	0.3737
End-stage Kidney disease	0 (0 %)	2 (1.07 %)	–	
Localized infection	3 (8.33 %)	13 (6.95 %)	0.693 (0.126–4.25)	0.6751
Malignancy	8 (22.22 %)	21 (11.23 %)	0.433 (0.099–1.617)	0.2170
Pneumonia/Lung disease	4 (11.11 %)	34 (18.18 %)	1.353 (0.28–6.543)	0.6979
Burn	0 (0 %)	7 (3.74 %)	–	
Sepsis	8 (22.22 %)	33 (17.65 %)	0.674 (0.158–2.457)	0.5582
Trauma	3 (8.33 %)	13 (6.95 %)	6.886 (1.362–39.351)	0.0208
Others	0 (0 %)	6 (3.21 %)	–	
**Invasive devices**				
Urinary Catheter	8 (22.22 %)	18 (9.62 %)	2.689 (1.007–6.678)	0.0482
Venous catheter	33 (91.66 %)	153 (81.81 %)	2.336 (0.771–10.534)	0.1444
Parenteral nutrition	9 (25.00 %)	15 (8.02 %)	3.808 (1.455–9.555)	0.0074
Mechanical ventilation	12 (33.33 %)	71 (37.96 %)	0.8221 (0.373–1.726)	0.6105
**No previous exposure to carbapenem antibiotics**	23 (63.88 %)	71 (37.96 %)	2.469 (1.182–5.361)	0.0159
**Previous exposure to carbapenem antibiotics**	6 (16.66 %)	47 (25.13 %)	0.6079 (0.214–1.470)	0.2831

CPE: carbapenemase producing *Enterobacterales*.

Several variables were analyzed in a comparison between CPE-positive and CPE-negative patients. Demographically, males represented 69.44 % of the CPE-positive group compared to 50.80 % in the CPE-negative group. This study found a statistically significant likelihood (OR = 2.18, p = 0.0412) of males being CPE positive. The average ICU stay was also considered longer for CPE-positive, with a duration of 22.5 days, compared to 15.6 days for CPE-negative individuals, marking a significant difference (p = 0.0002). Additionally, trauma was distinctly associated with CPE positivity. Patients with trauma were approximately 6.89 times more likely to be CPE positive (p = 0.0208) (Table 1).

Regarding invasive devices, this study showed differences in the usage of urinary catheters and parenteral nutrition. There was a higher incidence of urinary catheter use in CPE-positive patients (22.22 % vs. 9.62 %, OR = 2.689, p = 0.0482) and of parenteral nutrition (25.00 % vs. 8.02 %, OR = 3.808, p = 0.0074). Interestingly, 63.88 % of CPE-positive patients had not previously been exposed to carbapenem antibiotics, compared to 37.96 % in the CPE-negative group (OR = 2.469, p = 0.0159) (Table 1).

### 3.2 Bacterial identification and antimicrobial susceptibility

A total of 40 strains of CPE were isolated from rectal swabs of 36 patients with positive cultures. Among these, *K. pneumoniae* was the predominant species isolated, accounting for 52.5 % (n = 21) of the dataset. *Escherichia coli* constituted around 12.5 % (n = 5), *Enterobacter cloacae* represented 10 % (n = 4), *Klebsiella oxytoca* and *Klebsiella aerogenes* each accounted for 5 % (n = 2). In addition, other species, including *Proteus mirabilis, Serratia marcescens*, *Providencia rustigianii*, and various *Citrobacter* species, each accounted for a minimum of 2.5 % (n = 1) (Supplementary Fig. 1).

Since *Klebsiella pneumoniae* was the most frequently isolated bacterium, its antimicrobial susceptibility was summarized (Supplementary Fig. 2). Due to the production of carbapenemases, extensive resistance to beta-lactams was observed, including piperacillin-tazobactam, meropenem, imipenem, ertapenem, ceftriaxone, cefepime, and ampicillin. Variable susceptibility to aminoglycosides was noted, with 66.6 % susceptibility to gentamicin and 100 % susceptibility to amikacin. Additionally, 61.9 % of the isolates were susceptible to ceftazidime-avibactam.

### 3.3 Carbapenemase identification

**Fig. 1:**
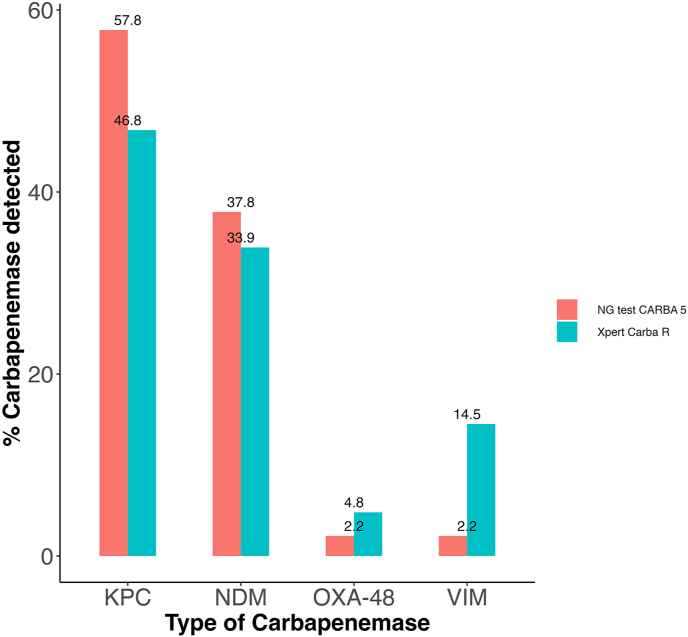
Molecular identification of several types of carbapenemases using two different methods. NG test CARBA 5 and Xpert Carb R were used to detect diverse types of carbapenemases like KPC, NDM, OXA-48, IMP, and VIM. Both tests show similar efficacy.

CPE bacterial colonies were assessed to detect the types of carbapenemases through the NG-test CARBA 5. Most carbapenemases detected were KPC (57.8 %) and NDM (37.8 %), with OXA-48, and VIM each at 2.2 %. Rectal swab specimens tested positive for CPE by phenotypic methods were then subjected to the Xpert Carba-R test. The percentages of carbapenemases varied regarding NG-test CARBA 5, with the detection of KPC (46.8 %), NDM (33.9 %), OXA-48 (4.8 %) and VIM (14.5 %). No IMP was found by either method (Fig. 1). Within the bacterial species, *K. pneumoniae* exhibits the highest prevalence of carbapenemases. Notably, KPC was the most frequent enzyme across all species listed (Table 2). The second most common enzyme was NDM. The co-production of carbapenemases was a rare phenomenon, but when present, it was predominantly between KPC and NDM (n = 6). The species *Citrobacter freundii* was unique in its co-production of VIM and NDM (n = 1) (Table 2).

**Table 2:** Types of carbapenemases of CPE isolates according to NG-Test Carba 5.

Species	CPE						
	Carbapenemase genotype					Carbapenemase co-production	
	KPC	NDM	VIM	OXA-48	IMP	KPC + NDM	VIM + NDM
*Klebsiella pneumoniae* (n = 21)	16	8	0	0	0	4	0
*Escherichia coli* (n = 5)	3	2	0	1	0	1	0
*Klebsiella oxytoca* (n = 2*)*	1	2	0	0	0	1	0
*Enterobacter cloacae* (n = 4)	3	0	0	0	0	0	0
*Klebsiella aerogenes* (n = 2*)*	1	1	0	0	0	0	0
*Proteus mirabilis* (n = 1)	0	1	0	0	0	0	0
*Citrobacter freundii* (n = 1)	0	1	1	0	0	0	1
*Citrobacter koseri* (n = 1)	1	0	0	0	0	0	0
*Citrobacter farmeri* (n = 1)	0	1	0	0	0	0	0
*Providencia rustigianii* (n = 1)	0	1	0	0	0	0	0
*Serratia marcescens* (n = 1)	1	0	0	0	0	0	0

CPE: carbapenemase producing *Enterobacterales*, KPC: *Klebsiella pneumoniae* carbapenemase, NDM: New Delhi metallo-betalactamase, VIM: Verona integron-encoded metallo-betalactamase, IMP: imipenemase metallo-β-lactamase, and OXA-48: oxacillinase-48.

## 4 Discussion

Recently, carbapenemase-producing *Enterobacterales* have spread globally, representing a significant threat to public health. In 2017, the World Health Organization (WHO) published the *'Global Priority List of Antibiotic-Resistant Bacteria'* to guide research, discovery, and the development of new antibiotics, issuing a critical alarm about CPE [15]. The epidemiological report of carbapenemases in Latin America (LATAM) and the Caribbean, updated until 2019, shows that *Enterobacterales,* *Pseudomonas spp*., and *Acinetobacter spp*. are the leading carriers of carbapenemases [16]. The prevalence of CPE isolation in ICUs among these countries varies significantly. Argentina accounts for a 26 % prevalence [17], while Ecuador has a higher prevalence of 37.67 % [18]. In this study, we report a lower prevalence of 16.12 %.

Several variables have been associated with CPE colonization and infection. These variables include prolonged hospitalization, invasive devices (such as orotracheal tubes, central venous catheters, and urinary catheters), gastrointestinal disease, invasive procedures, and prior exposure to broad-spectrum antibiotics [19]. An observational study in Cali, Colombia, revealed that a history of infection within the past year, liver disease, and the use of invasive devices were significant risk factors for colonization with MDR [20]. In this study, a significant association was found between CPE isolation and ICU stay (p = 0.0002), previous trauma (p = 0.0208), the use of invasive devices (p = 0.0482), male sex (OR = 2.18, p = 0.0412), but not with age (Table 1). Interestingly, data from an international matched case-control study of CPE risk factors found no significant difference in male sex (p = 0.42) [20]. Furthermore, a study conducted in Ecuador observed a significant association between CPE infection and burn patients [18]; in contrast, this study found a clear association with the admission diagnosis of trauma. Previous reports from Colombia have also highlighted significant associations with the use of venous catheters [21], and here a significant association was found with urinary catheters (p = 0.0482) and parenteral nutrition (p = 0.0074). These findings emphasize the importance of active surveillance and consider removing such invasive devices when appropriate.

Local information is crucial for implementing active surveillance programs for patients with asymptomatic CPE colonization. Combining this screening with interventions such as patient isolation, cohorts, environmental cleaning, and staff education could reduce infection and cross-infection rates [22]. There is a clear association between CPE infections and high mortality rates (40–50 %), with evidence showing a higher prevalence in inpatient facilities compared to the community [23]. In the analysis of mortality in our CPE-positive patients, the rate was 16.66 %, with no statistically significant difference compared to CPE-negative patients, which could be related to the low number of CPE-isolated in this study.

In Colombia, *K. pneumoniae* is the predominant pathogen detected in ICUs, with up to 14.6 % of isolates exhibiting carbapenem resistance [9]. Within this study, *K. pneumoniae* emerged as the species most associated with carbapenemase production, accounting for 52.5 % of cases. It is crucial to emphasize that according to the finding of the lateral flow test, the predominant carbapenemases among all species were KPC (57.8 %) and NDM (37.8 %), with no detection of IMP, and only a few reports of OXA-48 (2.2 %) and VIM (2.2 %). Similar results were obtained through molecular test (Fig. 1). These findings are like other reports in Colombia where KPC (70.3 %) and NDM (7 %) have also been reported as the most frequently occurring carbapenemases in *Enterobacterales* [9]. This observation also aligns with the findings of García-Betancur et al. from the Antimicrobial Resistance and Hospital Epidemiology Research Group (RAEH) in Colombia and other regions across Latin America [16].

The first report of a *K. pneumoniae* co-harboring carbapenemases VIM + KPC in Colombia occurred in 2013 [24]. Since then, there has been an increase in isolation reports of *Enterobacterales* that simultaneously produce more than one type of carbapenemase, such as VIM + KPC or NDM + KPC, and even triple combinations like KPC + NDM + VIM [25]. The most prevalent report of the coproduction of carbapenemases is KPC + NDM [24]. The emergence of multi-carbapenemase producers among *Enterobacterales* has increased in the post-COVID era (after 2019–2020), with cases of coproduction rising from 13 to 18 cases per year to 230 cases in 2023 (more than tenfold) [26]. In our short follow-up period of 10 months, we found six cases of KPC + NDM isolation in *K. pneumoniae*, *E. coli*, and *K. oxytoca*, and one case of VIM + NDM (Table 2). These findings have also been observed in other countries, such as Italy. Indeed, multi-carbapenemase producers were found in 0.8 % of CPE isolates, showing combinations such as KPC + VIM, NDM + OXA-48-like, and VIM + OXA-48-like dual-carbapenemases [27]. This is a concerning public health issue due to the rapid emergence of new resistance mechanisms and the lack of new antimicrobial drugs in Latin America.

Multiple studies have demonstrated changing trends in antimicrobial resistance among isolated *K. pneumoniae* [28]. A study conducted in Italy revealed antibiotic susceptibility patterns, indicating that more than 50 % of the isolates exhibited resistance to cephalosporins, fluoroquinolones, and penicillin, and 20 %–40 % of *K. pneumoniae* were resistant to carbapenems and aminoglycosides [29]. The antimicrobial susceptibility of *K. pneumoniae* in this study showed susceptibility rates of 100 % and 61.9 % for amikacin and ceftazidime-avibactam, respectively. Considering the emerging resistance to dual therapies like ceftazidime/avibactam (resistant in 38.1 % of *K. pneumoniae* of this study, Supplementary Fig. 2), other treatment options that are not available in Colombia, such as imipenem/cilastatin/relebactam, cefiderocol, and meropenem/vaborbactam, are increasingly needed in our country. Also, novel drugs like plazomicin, eravacycline, and imipenem/relebactam, currently part of antibiotic stewardship [30], represent international needs as treatment options for these microorganisms [31].

Regarding the methods for identifying carbapenemases, there are some technical limitations because no laboratory test has 100 % sensitivity and specificity in detecting the carbapenemase families and their known alleles [6]. Additionally, Some carbapenemase variants are not identified with tests like NG-Test CARBA 5 and Xpert Carba-R, that only detect the most frequent five types of enzymes (KPC, NDM, VIM, IMP and OXA-48). Besides, lateral flow immunoassays may not detect KPC variants with mutations in the omega loop associated with ceftazidime/avibactam resistance [32,33]. Several studies have compared the performance of lateral flow test NG-Test CARBA 5 and molecular test Xpert Carba-R [[34], [35], [36], [37]]. According to a recent study, NG-Test CARBA 5 has a sensitivity of 100 % and a specificity of 99 %, while Xpert Carba-R has a sensitivity of 99.8 % and a specificity of 100 % [34]. This study found a comparable performance between NG-Test CARBA 5 and Xpert Carba-R. However, differences in the frequency of carbapenemase detection were observed. In some cases, several carbapenemases were detected in one sample with the Xpert Carba-R compared to the NG-Test CARBA 5, suggesting that a patient could harbor multiple microorganisms producing different carbapenemases. These differences could be attributed to the type of sample used for each test, bacterial colonies for the NG-Test CARBA 5, and rectal swab samples for Xpert Carba-R.

It is important to note that this research spanned a modest ten-month follow-up period, with some limitations, like the absence of continuous surveillance in our testing process. Rectal swabs were taken from each patient after they had spent seven or more days in the ICU, making it challenging to distinguish between patients previously colonized/infected with CPE and those who became colonized/infected during their stay. There were no outbreaks of microorganisms in the hospital during the development of this study, and we adhered to the national guidelines suggested by the National Institute of Health of Colombia to prevent the spread of multidrug-resistant organisms [38]. As a significant limitation, we did not conduct follow-ups on patients on the day of admission to the ICU, 48 h, and the days later; only a single test was performed during their stay. Additionally, we observed no significant association with mortality in CPE-colonized/infection cases due to the small proportion of isolations, and despite testing 223 patients, we found a low rate of positivity among them.

## 5 Conclusion

This study shows a low prevalence of CPE in ICUs, specifically 16.12 %, with a significant predominance of *K. pneumoniae* carrying KPC carbapenemase. Among the different types of carbapenemases, KPC was the most prevalent with 57.8 %, followed by NDM with 46.8 %. Identifying distinct carbapenemase subtypes provides valuable information on dissemination pathways and contributes to strengthening efforts to manage containment within ICUs in Cali, Colombia.

## Data availability

Data will be made available upon reasonable request.

## Funding

This work was supported by internal inter-institutional call from Universidad del Valle-10.13039/501100009543Pontificia Universidad Javeriana Cali 2021. Universidad del Valle No. 1940, Pontificia Universidad Javeriana Cali No. 020100774.

## Ethics approval

This study was reviewed and approved by the Research Ethics Committee of Universidad del Valle (approval No. 003–022), the Tertiary Hospital in Cali (approval No. 007–2022), and Pontificia Universidad Javeriana Cali (approval No. 010–2021).

## Consent to participate

Informed consent was obtained from all individual participants included in the study.

## CRediT authorship contribution statement

**Mónica Fernandes-Pineda:** Writing – original draft, Methodology, Investigation. **Ernesto Martínez-Buitrago:** Writing – review & editing, Methodology, Funding acquisition, Conceptualization. **José H. Bravo:** Writing – review & editing, Supervision, Funding acquisition. **Lorena Matta-Cortés:** Writing – review & editing, Supervision, Formal analysis. **Johann A. Ospina-Galindez:** Writing – review & editing, Formal analysis, Data curation. **Claudia C. Paredes-Amaya:** Writing – review & editing, Supervision, Methodology, Formal analysis.

## Declaration of competing interest

The authors declare that they have no known competing financial interests or personal relationships that could have appeared to influence the work reported in this paper.
