# Supplementary material for: Characterization of carbapenemase-producing Enterobacterales from rectal swabs of patients in the intensive care units of a tertiary hospital in Cali-Colombia
Source: Heliyon. 2024 Jun 21;10(12):e33368. doi: 10.1016/j.heliyon.2024.e33368 (PMC11254587; doi:10.1016/j.heliyon.2024.e33368)

**Characterization of Carbapenemase-producing *Enterobacterales* from rectal swabs of patients in the intensive care units of a tertiary hospital in Cali-Colombia**

Mónica Fernandes-Pineda^a^, Ernesto Martínez-Buitrago^a^, José H. Bravo^b^, Lorena Matta-Cortés^a^, Johann A. Ospina-Galindez^c^, Claudia C. Paredes-Amaya^d*^

^a^ Department of Internal Medicine, Faculty of Health, Universidad del Valle, Cali, Colombia.

^b^ Department of Medical Sciences, Faculty of Health Sciences, Pontificia Universidad Javeriana Cali, Cali, Colombia

^c^ Faculty of Engineering, Universidad Autónoma de Occidente, Cali, Colombia

^d^ Department of Microbiology, Faculty of Health, Universidad del Valle, Cali, Colombia

*Corresponding author: Department of Microbiology, Faculty of Health, Universidad del Valle, Cl. 4b #36-00, Cali, Colombia. E-mail: [claudia.paredes@correounivalle.edu.co](mailto:claudia.paredes@correounivalle.edu.co)

**Supplementary material**

**Figure. 1 Distribution of bacterial species isolated** *Klebsiella pneumoniae* is the predominant specie of the dataset (52.5%), following by *Escherichia coli* (12.5%), Enterobacter *cloacae* (10%), and *Klebsiella oxytoca* (5%). Conversely, several species, including *Serratia marcescens*, *Providencia rustigianii*, and various *Citrobacter* species, each account for a minimal 2.5%


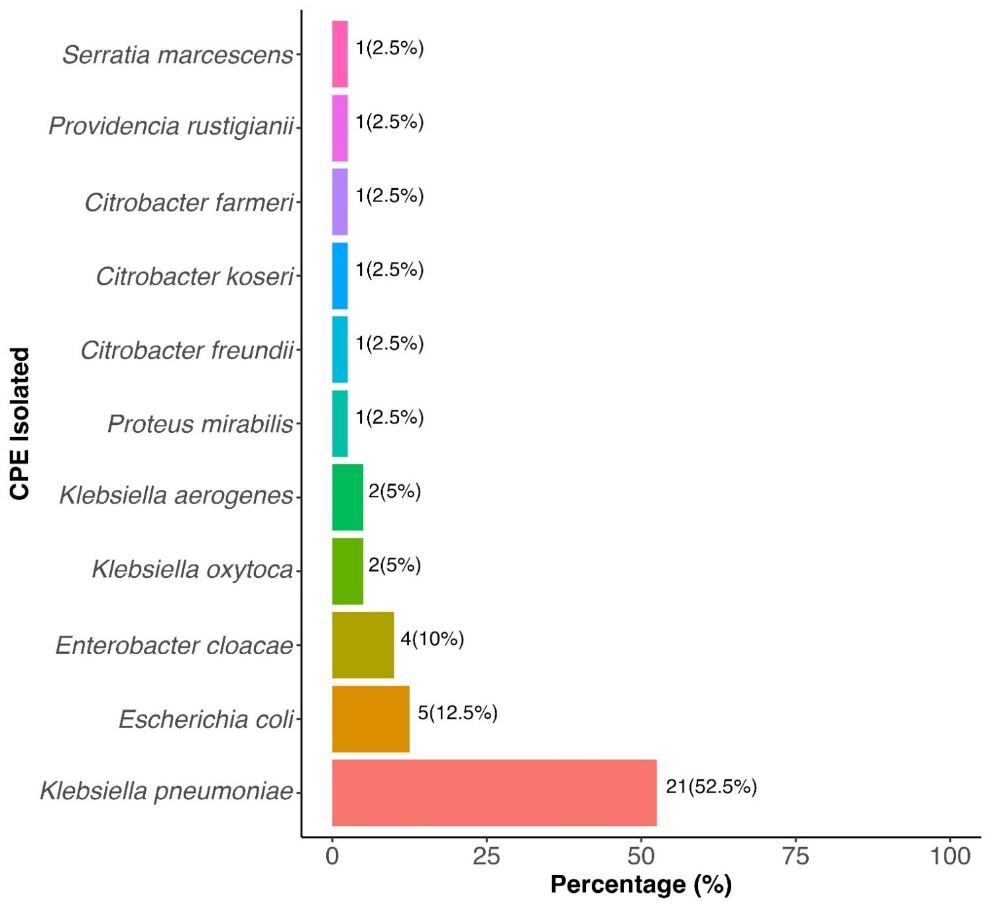


**Figure 2. Antimicrobial susceptibility of *Klebsiella pneumoniae***


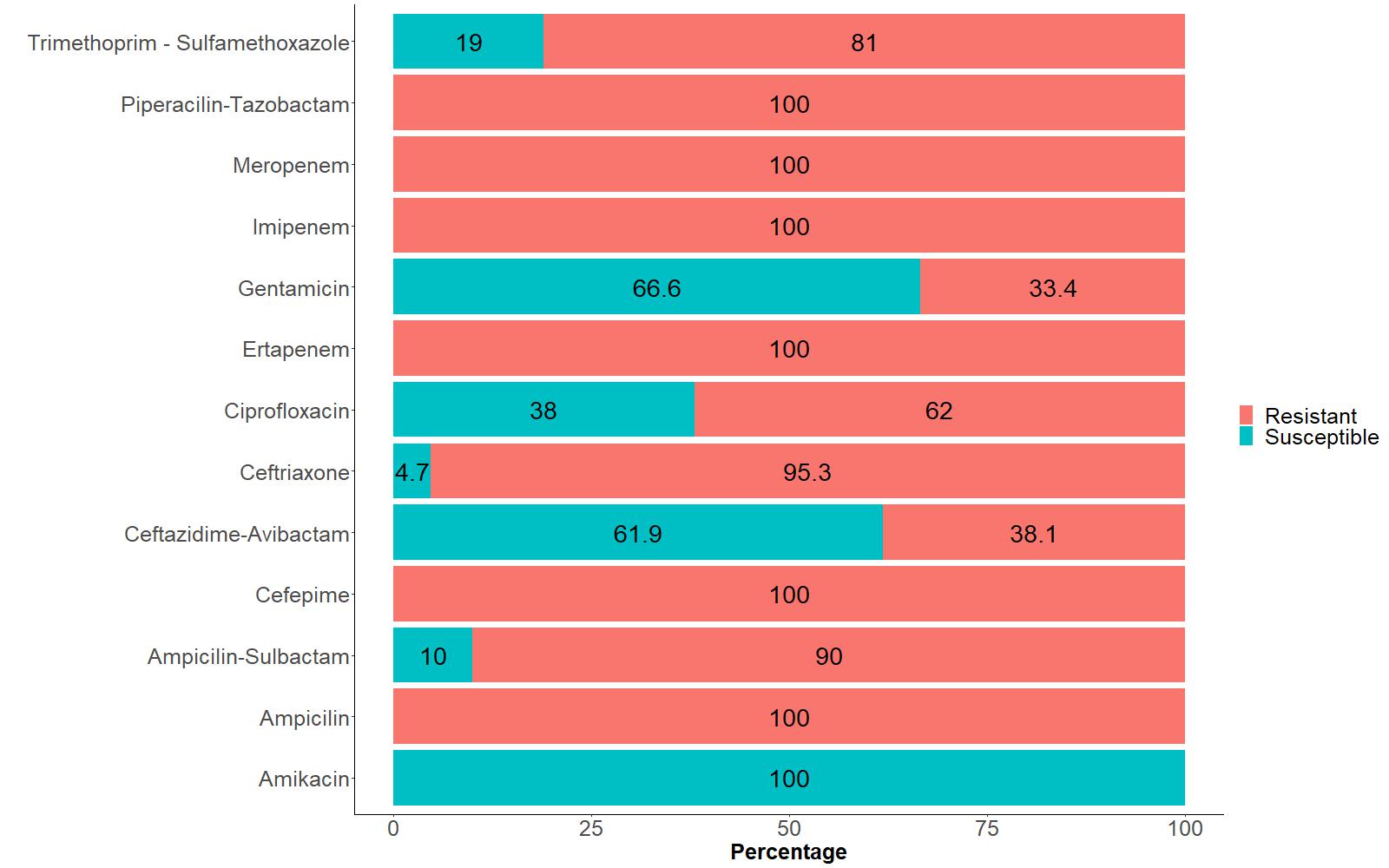

Supplement: Multimedia component 1 [file mmc1.docx]
